# Supplementary material for: Getting More Out of Biomedical Documents with GATE's Full Lifecycle Open Source Text Analytics
Source: PLoS Comput Biol. 2013 Feb 7;9(2):e1002854. doi: 10.1371/journal.pcbi.1002854 (PMC3567135; doi:10.1371/journal.pcbi.1002854)
Supplement: Dataset S1 — GATE software. Dataset S1 bundled with this paper contains a distribution of GATE (or see http://gate.ac.uk/download/). (TGZ) [file pcbi.1002854.s001.tgz › plos-gate/plugins/Parser_RASP/doc/index.html]

RASP2 plugin for GATE

  

# RASP2 plugin for GATE

Next: Modules

## Overview

This
resource is
an integration of the RASP
System into the GATE
framework.  
  
RASP is a
domain-independent, robust parsing system for English. For
ease of installation, the system is distributed in the form of binaries
for 3 widespread unix architectures (Intel-32bit and -64bit/Linux, and
Sparc/Solaris). It is free for
research and evaluation purposes. RASP is described in :  
  

|  |  |
| --- | --- |
|  | Briscoe, E., J. Carroll and R. Watson (2006) The Second Release of the RASP System. In *Proceedings of the COLING/ACL 2006 Interactive Presentation Sessions*, Sydney, Australia. |

  


GATE
is an infrastructure for developing and deploying software components
that process human
language. GATE helps scientists and developers in three ways:

1. by speciﬁying an architecture, or organisational
   structure, for language processing software;
2. by providing a framework, or class library,
   that implements the architecture and can be used to embed language
   processing capabilities in diverse applications;
3. by providing a development environment built
   on top of the framework made up of convenient graphical tools for
   developing components.

Please contact the respective projects for any question
related to RASP or GATE. You can use the DigitalPebble
user group for any question specific to this plugin.

## Installation

RASP   
  
RASP needs to be installed. Please note that RASP is available only on Unix, Linux or Mac
platforms. In the rest of this document we'll assume that
RASP has been installed on /usr/local/bin/RASP2.  
  
First go to the directory where you've installed RASP and
enter the following command :  
  
echo "This is a test" |
./scripts/rasp.sh"  
  
after a few seconds you should get something similar to :  
  
(|This:1\_DD1|
|be+s:2\_VBZ| |a:3\_AT1| |test:4\_NN1|) 1 ; (-4.902)  
  
(|ncsubj| |be+s:2\_VBZ|
|This:1\_DD1| \_)  
(|xcomp| \_ |be+s:2\_VBZ|
|test:4\_NN1|)  
(|det| |test:4\_NN1|
|a:3\_AT1|)  
  
If this is not the case, check that you've installed RASP properly and
that it is available for your platform.  
  
RASP2 plugin  
  
The
plugin is provided as a jar file compiled with Java 1.5. However, if
you need to make changes or compile it with a different version, you
can use the ANT script  to recompile the source code.   
  
The
RASP2 plugin needs to be enabled from the plugin console in the GATE
GUI. Check the GATE documentation to see how to do that.  
  
The plugin consists in a number or modules which are detailed in the following section.  
  
  
  
 
